# Dietary Patterns and the Social Development Index in Mexico City: A Machine-Learning Approach

**DOI:** 10.3390/nu18183100

**Published:** 2026-09-21

**Authors:** Mireya Martínez-García, Guadalupe Gutiérrez-Esparza, María del Carmen González Salazar, Luis M. Amezcua-Guerra, Enrique Hernández-Lemus

**Affiliations:** 1 Department of Immunology, National Institute of Cardiology Ignacio Chávez, Mexico City 14080, Mexico; lmamezcuag@gmail.com; 2 Department of Dental Public Health, Graduate Studies and Research Division, School of Dentistry, Universidad Nacional Autónoma de México, Mexico City 04510, Mexico; 3 ”Researcher for Mexico” Program Under SECIHTI, Secretariat of Sciences, Humanities, Technology, and Innovation, Mexico City 08400, Mexico; ggutierreze@secihti.mx; 4 Diagnostic and Treatment Services, National Institute of Cardiology Ignacio Chávez, Mexico City 14080, Mexico; 5 Department of Nutrition, Ambulatory Care Services, National Institute of Cardiology Ignacio Chávez, Mexico City 14080, Mexico; carmen.gonzalez@cardiologia.com; 6 Computational Genomics Division, National Institute of Genomic Medicine, Mexico City 14610, Mexico

**Keywords:** dietary habits, urban populations, territorial analysis, social determinants of nutrition, food consumption

## Abstract

Background: Dietary composition reflects the interplay of individual and contextual factors, yet its relationship with socioeconomic–territorial conditions remains insufficiently characterized in large Latin American cities. Methods: We identified empirical dietary patterns among 3439 adults from the cohort in Mexico City and examined their association with the Social Development Index (SDI), a composite area-level measure of socioeconomic–territorial development. Energy-adjusted food-group intakes were analyzed using k-means clustering, and supervised machine-learning models with SHAP-based interpretation were used to characterize pattern membership. Results: Three dietary patterns—Fresh, Mixed, and Refined—were identified along a continuous dietary gradient primarily differentiated by the relative contribution of fruits and vegetables. SHAP analysis showed that the Fresh pattern was characterized mainly by female sex and older age, the Refined pattern by greater contributions from physical activity, cardiometabolic markers, male sex, and lower SDI values, whereas the Mixed pattern was distinguished primarily by cardiometabolic variables, alcohol intake, and durable-goods ownership. Dietary pattern membership was significantly associated with SDI stratum ($\chi^{2}=40.77$, p<0.001), although the effect size was small (Cramér’s V = 0.078). The proportion of participants classified in the Fresh pattern increased from 35.7% in the lowest SDI stratum to 51.9% in the highest, whereas the Refined pattern decreased from 44.6% to 34.0%. Supervised models showed limited discriminatory performance (balanced accuracy ≈ 0.40), indicating substantial overlap among patterns despite their distinct multivariable profiles. Conclusions: These findings indicate that dietary variation in this urban population is better represented as a continuum than as sharply separated dietary phenotypes.

## 1. Introduction

Dietary patterns aim to capture the overall dietary profile of individuals by encompassing the types of food and beverages consumed, the portion sizes, the frequency of consumption and the different ways in which these are combined. Over the last two decades, the analysis of dietary patterns has emerged as a complementary approach to the study of individual nutrients or food items, providing a more comprehensive understanding of dietary behaviors in diverse populations [1,2,3]. Several dietary patterns have been consistently identified in nutritional epidemiology [4,5]. Among the reported patterns are those that have been labeled as Western, prudent or healthy, DASH or Mediterranean, balanced, traditional, high in meat, plant-based, vegetarian or vegan, snacking, and ultra-processed [6,7,8,9,10].

Dietary patterns are commonly assessed using two main approaches: index-based methods (a priori), which rely on predefined dietary scores that evaluate overall diet quality, based on nutritional guidelines and the presumed health effects of specific dietary components; and data-driven (or a posteriori) methods, which apply multivariate statistical techniques—such as factor analysis, principal component analysis (PCA), reduced rank regression (RRR), latent class analysis (LCA) or cluster analysis—to identify patterns directly from dietary intake data independently of prior health assumptions [4,11,12]. Dietary pattern analysis allows researchers to capture the complexity of the diet and its interaction with sociocultural, economic, and environmental factors [13,14,15,16].

Mexico is an upper-middle-income country undergoing an advanced nutrition transition while experiencing persistent socioeconomic and territorial inequalities that influence dietary behaviors and health outcomes [17,18,19]. Mexico City, the country’s capital and the core of the Metropolitan Area of the Valley of Mexico, is one of the largest urban agglomerations in the world, with more than 25 million inhabitants distributed across highly heterogeneous social and built environments [20,21]. This marked territorial diversity makes Mexico City an appropriate setting for investigating how empirical dietary patterns relate to contextual measures of social development.

Socioeconomic inequalities in Mexico have historically shaped dietary and lifestyle behaviors, influencing access to healthy foods and contributing to disparities in nutrition-related health outcomes [22,23,24]. Although previous studies have examined the relationship between socioeconomic status and dietary patterns using individual-level indicators, relatively little attention has been given to area-based measures that capture the broader social context in which dietary behaviors develop [25,26]. The Social Development Index (SDI), developed by the Government of Mexico City, is a multidimensional territorial indicator that integrates information on education, housing, and access to essential goods and services to characterize social inequalities across neighborhoods [27]. Previous studies have shown that the SDI provides a useful framework for examining how structural social conditions relate to health and dietary outcomes [28,29,30].

Against this background, the present study uses baseline data from the Tlalpan 2020 cohort [31], a population-based prospective study designed to investigate cardiometabolic risk factors among adults residing in Mexico City. We applied an unsupervised machine-learning approach to identify empirical dietary patterns within this urban population and subsequently characterized these patterns according to their dietary composition and their sociodemographic, lifestyle, anthropometric and clinical profiles. We also evaluated their relationship with the SDI of participants’ place of residence and explored the contribution of individual-level characteristics to dietary pattern discrimination using supervised machine-learning models with SHAP-based interpretation. Although the Tlalpan 2020 cohort has generated numerous studies on cardiometabolic risk factors and lifestyle, this is the first analysis to investigate empirical dietary patterns within the cohort and examine their relationship with socioeconomic–territorial context using an integrated machine-learning approach.

## 2. Materials and Methods

### 2.1. Study Design and Population

The present work is a cross-sectional data analytics study. The Tlalpan 2020 cohort includes clinically healthy men and women, 20 to 50 years of age residing in Mexico City, Mexico [31]. Eligible participants were those with complete baseline data on dietary intake, clinical and sociodemographic characteristics. Individuals with implausible energy intake values (≤500 or ≥5000 kcal/day) were excluded from our analyses.

### 2.2. Ethical Considerations

The present study is a secondary cross-sectional analysis of baseline data from the Tlalpan 2020 cohort. All participants were provided written informed consent before enrollment. The Tlalpan 2020 cohort was conducted in accordance with the Declaration of Helsinki and was approved by the Research Ethics Committee of the National Institute of Cardiology Ignacio Chávez (INCICH) under protocol code 13-802 [31]. Ethical approval was renewed in 2020 to continue cohort follow-up and allow subsequent secondary analyses.

### 2.3. Data Collection

Baseline data used in the present analysis were collected between 2014 and 2019 as part of the Tlalpan 2020 cohort [31]. Information was obtained through structured interviews and standardized physical assessments performed by trained personnel, including dietary intake, sociodemographic and socioeconomic–territorial characteristics, lifestyle factors (physical activity, sleep quality, psychological stress, alcohol intake, energy drink consumption, and smoking status), anthropometric measurements, and predefined biochemical markers [31,32].

#### 2.3.1. Dietary Intake

Dietary intake was assessed using a validated Semi-quantitative Food Frequency Questionnaire (SFFQ) developed by the National Institute of Public Health (INSP, Cuernavaca, Morelos, Mexico) for the Mexican adult population [33]. Energy and nutrient intakes were estimated using the Nutritional Habits and Nutrient Consumption Evaluation System (SNUT, version 3.0; National Institute of Public Health, Cuernavaca, Morelos, Mexico) and the Mexican System of Equivalent Foods (SMAE, 5th ed.)  [34,35].

Participants reported their usual dietary intake during the previous year. The SFFQ included 104 food and beverage items with predefined portion sizes covering major food groups, including dairy products, fruits, natural fruit juices, eggs, meats and processed meats, fish and seafood, fresh vegetables, legumes, refined grains, whole grains or high-fiber cereals, sweets and desserts, beverages, fats and spreads, maize products, and traditional Mexican foods [36]. Additional questions assessed the habitual use of sugar and salt, consumption of chicken skin and visible meat fat, and the use of vitamin and calcium supplements.

Consumption frequency was recorded using ten standardized response categories ranging from never to ≥6 times/day. Reported frequencies were converted into daily consumption frequencies using standardized conversion factors (e.g., 2–4 times/week = 0.43 times/day) [37]. Daily food intake (g/day) was then estimated by multiplying the daily consumption frequency by the corresponding standardized portion size. Individual food intakes were subsequently aggregated into food groups and expressed as energy-adjusted densities (g/1000 kcal), which were used for all clustering analyses. Total energy intake was retained as a descriptive variable.

#### 2.3.2. Sociodemographic Characteristics

Sociodemographic data included age (in years), sex (men and women), marital status (single, married and other), educational attainment (highest educational level concluded), employment status (student, business executive, housekeeper, professional, manually qualifies, manually unqualified, other and unemployed), and geographic location of residence. As a contextual proxy for socioeconomic status, we used the SDI. The SDI is a composite socioeconomic–territorial indicator designed to measure the relative levels of social development in all the administrative territorial units of the Mexico City [27]. Each participant was geocoded based on their residential address (postal code and neighborhood) and linked to the corresponding territorial unit. SDI values for each unit were obtained from the publicly available dataset provided by the Council for the Evaluation of Social Development of Mexico City [38].

The SDI incorporates multiple dimensions of deprivation and opportunity, including quality and available space in the home (flooring type and the number of occupants per bedroom), educational access (completion of secondary education (≥13 years of schooling)), access to social security and/or Medical Service (coverage by any Mexican health system), durable goods (ownership of at least three household assets), sanitary adequacy (water supply, toilet facilities, drainage system) and electricity access (availability of electricity in the household) [30]. The index ranges from 0 to 1, with higher scores indicating more favorable social development conditions and lower scores reflecting greater social disadvantage. Based on the resulting value, levels of development are classified into four stratum: (1) very low, (2) low, (3) medium and (4) high [28].

#### 2.3.3. Lifestyle Behaviors

Additional covariates included physical activity, sleep quality and psychological stress to account for the potential influence of these features on dietary behavior and their broader role in shaping both lifestyle and health-related patterns [39,40,41,42,43].

Physical activity was measured using the long version of the International Physical Activity Questionnaire (IPAQ). The IPAQ is a widely used instrument that is known to have well-established reliability and it has been validated in diverse populations, including their use to survey Mexican adults. The IPAQ works by collecting information about the frequency and duration of vigorous, moderate, and walking activities spanning over the last seven days prior to surveying. Total physical activity was calculated in terms of the metabolic equivalent task minutes per week (MET-min/week) units [44].

Sleep quality was assessed using the Spanish version of the Medical Outcomes Study Sleep Scale (MOS-SS). The MOS-SS is also a validated instrument that evaluates sleep characteristics during the previous four weeks [45]. The scale measures multiple dimensions of sleep, including sleep duration (SLPQRAW), adequacy (SLPA), somnolence (SLPS), sleep awakening short of breath or with headache (SLPSOB), snoring (SLPSNR) and sleep disturbances (SLPD). Scores for each domain range from 0 to 100, with higher values indicating a greater presence of the characteristic assessed. The I-index, a global Sleep Problems Index was also calculated. Higher I-index scores reflect an individual’s poorer overall sleep quality and greater sleep impairment [46,47].

Psychological stress was analyzed using the Spanish version of the State–Trait Anxiety Inventory (STAI). The STAI is a psychometric instrument widely used to measure both state and trait anxiety by calculating STAI-S, a temporary indicator, and STAI-T, a chronic indicator. The version of STAI that has been adapted and validated for Mexican and Spanish-speaking populations was the one we used [48]. This implementation provides reliable quantification of psychological distress. Self-reported smoking status, alcohol intake and energy drink consumption were evaluated using standardized questionnaires.

#### 2.3.4. Clinical and Biochemical Measurements

Anthropometric measurements were obtained following standardized protocols. Body weight, height, and waist circumference were measured using clinical-grade calibrated equipment. Raised waist circumference was defined as ≥90 cm in men and ≥80 cm in women, following the International Diabetes Federation criteria for Latin American populations. Resting heart rate and blood pressure were measured using standardized procedures. Hypertension was defined as systolic blood pressure ≥140 mmHg and/or diastolic blood pressure ≥90 mmHg following current international guidelines [31,49].

Biochemical parameters were assessed from fasting blood samples (12 h fast) and 24 h urine collections. Analyses were performed at the accredited Central Laboratory of the INCICH using standardized automated methods (accreditation NMX-EC-15189-IMNC-2015/ISO 15189:2012 standard number CL-137). Serum measurements included fasting glucose, total cholesterol, triglycerides, low-density lipoprotein cholesterol (LDL-C), high-density lipoprotein cholesterol (HDL-C), uric acid, creatinine, and sodium. The atherogenic index was calculated as the LDL-C/HDL-C ratio. The 24 h urine samples were analyzed to determine levels of urinary sodium, potassium, and creatinine. Collection completeness was verified using sex-specific creatinine excretion reference ranges. All of our laboratory procedures followed standardized quality-control protocols [31].

### 2.4. Analytical Methods

**Figure 1:**
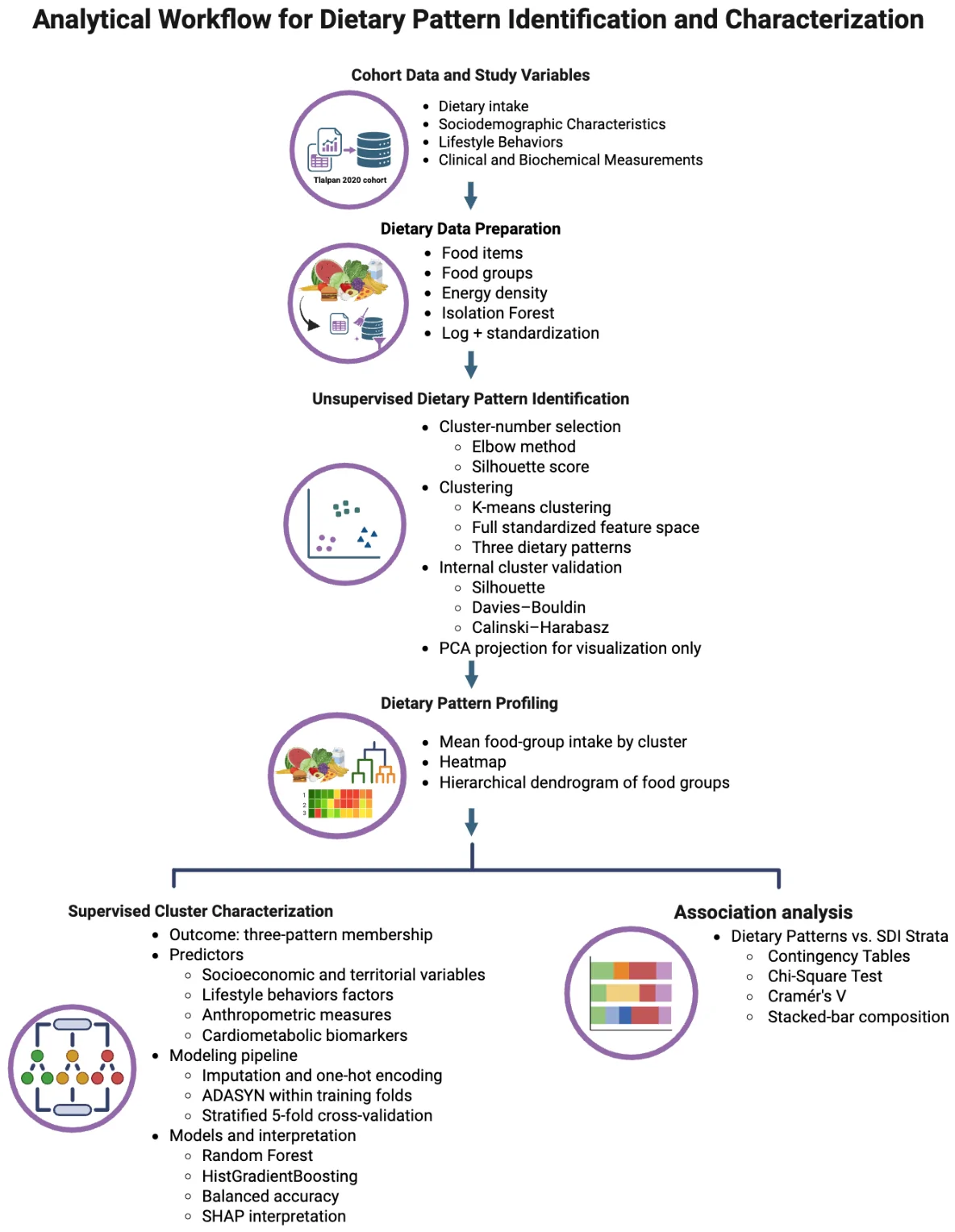
Analytical workflow for the identification and characterization of dietary patterns and their relationships with individual-level characteristics and the Social Development Index. Created in BioRender. Hernandez-Lemus, E. (2026) https://BioRender.com/5nmw8nt.

A two-stage machine-learning framework was used to characterize dietary patterns and examine their relationships with behavioral, clinical, socioeconomic, and territorial factors. First, energy-adjusted food-group intakes were analyzed using unsupervised clustering to identify empirical dietary patterns without predefined labels. Second, supervised classification models were applied to evaluate the extent to which cluster membership could be characterized by non-dietary variables. The objective of this stage was not to predict dietary intake, but to assess the contribution of individual and contextual characteristics to the differentiation of the identified patterns. In a subsequent analysis, dietary pattern membership was examined across Social Development Index strata, which were used as a proxy for broader socioeconomic–territorial gradients. All analyses were performed using Python 3.9.13 with standard machine-learning libraries. Figure 1 presents a schematic walk through the full workflow.

#### 2.4.1. Dietary Data Preprocessing

The dietary data first underwent a preprocessing stage. Individual food items were aggregated into food groups by nutritional similarity, and each group was expressed as a density (grams per 1000 kcal) to remove the influence of total energy intake and capture dietary composition rather than the overall quantity of food consumed. This energy-adjusted (density) format is recommended for cluster analysis of dietary data [50,51]. As part of this preprocessing, extreme observations were identified and removed using the Isolation Forest algorithm, an ensemble-based anomaly detection method suitable for high-dimensional data and previously applied in nutritional and biomedical contexts [52]. The energy-adjusted variables were then log-transformed to reduce skewness and subsequently standardized, limiting the influence of residual outliers and ensuring comparability across food groups. The resulting preprocessed variables were then used as input for the clustering stage described below.

#### 2.4.2. Unsupervised Identification of Dietary Patterns

The optimal number of clusters was selected using a combination of the Elbow method and the Silhouette score, balancing within-cluster compactness and between-cluster separation [53]. Based on these criteria, K-means clustering was applied to classify participants into mutually exclusive dietary patterns. Clustering was performed in the full standardized feature space. Principal component analysis (PCA) was conducted post hoc solely for visualization purposes and did not influence cluster assignment.

Internal cluster validity was evaluated using multiple complementary metrics, including the global Silhouette score, the Davies–Bouldin index, and the Calinski–Harabasz index, which jointly assess cluster cohesion and separation [54,55]. Cluster compactness was further examined through mean intra-cluster distances. Cluster-level dietary profiles were characterized by computing mean food group intakes within each cluster and visualized using heatmaps and hierarchical dendrograms to facilitate interpretation of dietary gradients.

#### 2.4.3. Supervised Modeling of Dietary Pattern Membership

To evaluate the extent to which non-dietary characteristics could discriminate dietary pattern membership, supervised machine-learning models were implemented using dietary cluster membership as the target variable. This step was not intended to optimize individual-level prediction, but rather to evaluate how well non-dietary characteristics classified dietary pattern membership and to identify the variables contributing most strongly to model predictions.

Predictors comprised the non-dietary variables available in the cohort, grouped into four domains: demographic and anthropometric (age, sex, weight, height, body mass index, waist circumference, systolic and diastolic blood pressure, and heart rate); lifestyle behaviors (total physical activity in MET-min/week, state and trait anxiety, sleep-quality indicators, smoking status, alcohol intake, and energy drink consumption); cardiometabolic biomarkers (uric acid, creatinine, HDL and LDL cholesterol, total cholesterol, triglycerides, fasting glucose, and sodium); and socioeconomic–territorial characteristics (marital status, the individual Social Development Index value and its stratum, and the SDI component dimensions of education, housing quality, housing lag, sanitation, health-service access, durable-goods ownership, and energy adequacy). Dietary variables were deliberately excluded from the predictor set because they define the clustering outcome and their inclusion would render the classification circular. Numerical and categorical variables were preprocessed using a unified pipeline implemented with a ColumnTransformer, including median imputation for numerical variables and most-frequent imputation followed by one-hot encoding for categorical variables. All preprocessing steps were embedded within the modeling pipeline to prevent data leakage. Random Forest and HistGradientBoosting classifiers were then trained using this pipeline, with hyperparameters optimized by grid search under stratified five-fold cross-validation and balanced accuracy as the primary metric [56].

#### 2.4.4. Class Imbalance Handling and Model Comparison

The three-cluster dietary pattern membership derived from the unsupervised analysis served as the target variable for the supervised classification models. All three clusters were retained for model development and evaluation. To address residual class imbalance, Adaptive Synthetic Sampling (ADASYN) was applied exclusively to the training folds within an imblearn pipeline [57]. Both Random Forest and HistGradientBoosting classifiers were evaluated using the same hyperparameter optimization and validation strategy described above.

#### 2.4.5. Model Interpretability

Model interpretability was assessed using SHAP (Shapley Additive Explanations), a game-theoretic approach for explaining machine-learning predictions [58]. Because both classifiers showed comparable predictive performance, SHAP values were computed for the HistGradientBoosting model in the three-class setting. To ensure interpretability based on observed data while maintaining computational feasibility, SHAP analysis was conducted on a random subsample of 800 real participants from the preprocessed training set using the fitted preprocessing pipeline, explicitly excluding synthetic samples generated by ADASYN. Class-specific SHAP values were derived from predicted probabilities to identify predictors that increased or decreased the likelihood of membership in each dietary pattern.

To complement model-based interpretability, we constructed a composite psycho-behavioral risk index integrating sleep quality, anxiety, and risk-related behaviors (Table 1). Sleep quality was categorized into tertiles based on the sleep duration averaged (SLPQRAW), defining poor, intermediate, and good sleep. Anxiety was categorized into tertiles for both state and trait anxiety scores and combined into a global anxiety indicator (high anxiety if either dimension was high; low anxiety if both were low; otherwise moderate). Behavioral risk was defined as the presence of at least one of four binary behaviors (alcohol intake, energy drink consumption, current smoking, and passive smoking exposure). Each domain (sleep quality, anxiety, and behavioral risk) contributed one point to the composite index. The final index ranged from 0 to 3, reflecting the accumulation of adverse psycho-behavioral domains rather than individual behaviors.

#### 2.4.6. Association Between Dietary Patterns and Social Development Strata

Associations between dietary patterns and SDI strata were assessed using contingency tables and formally tested with the chi-square test of independence. Effect size was quantified using Cramér’s V to evaluate the magnitude of association independently of sample size [59]. Stacked-bar diagrams were used for visualization only, while inferential conclusions were based on statistical testing.

### 2.5. Main Analytical Variables

The principal analytical variable was dietary pattern membership, identified from participants’ usual dietary intake assessed with the SFFQ. The SDI was included as the main contextual variable. The SDI was used to examine the distribution of dietary patterns across different levels of social development through association analyses.

Additional variables used to characterize dietary pattern membership included sociodemographic characteristics, lifestyle behaviors, anthropometric measurements, and cardiometabolic biomarkers. These variables were incorporated into the supervised learning models to characterize the multidimensional profiles associated with each dietary pattern.

## 3. Results

**Figure 2:**
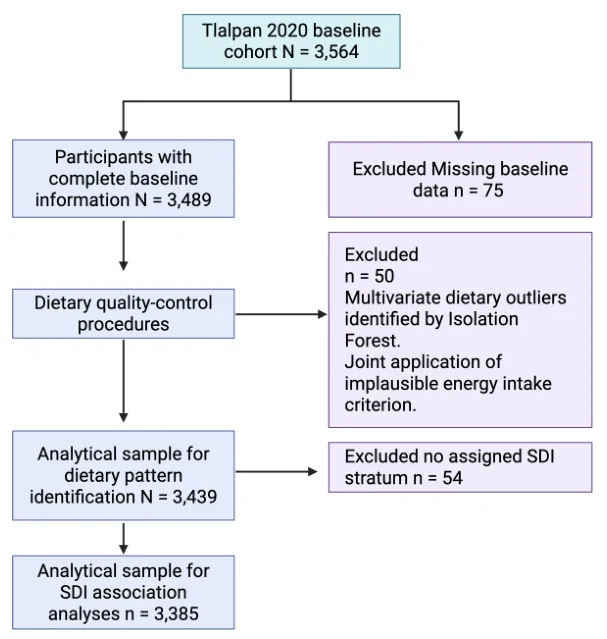
Flow diagram showing the derivation of the analytical samples from the Tlalpan 2020 baseline cohort. Created in BioRender. Hernandez-Lemus, E. (2026) https://BioRender.com/kslpg2r.

The derivation of the analytical sample is presented in Figure 2. Of the 3564 participants enrolled in the Tlalpan 2020 baseline cohort, 75 were excluded because of missing baseline data, leaving 3489 participants with one complete baseline observation per individual. Multivariate dietary outliers identified using the Isolation Forest algorithm (1% contamination) and participants with implausible energy intake were subsequently excluded through the joint application of both criteria (n=50), of whom 35 were identified as dietary outliers, yielding a final analytical sample of 3439 participants for dietary pattern identification. Among these, 3385 participants had an assigned Social Development Index stratum and were included in the SDI association analysis.

The analytical sample had a mean age of 37.5±9.1 years, and 62.6% of participants were women. Anthropometric and cardiometabolic characteristics stratified by sex are presented in Supplementary Table S1a. Men exhibited higher median BMI, waist circumference, systolic and diastolic blood pressure, LDL cholesterol, triglycerides, and fasting glucose, whereas women showed higher HDL cholesterol concentrations. Overweight and obesity were also more prevalent among men. Regarding the socioeconomic–territorial context, 52% of participants resided in neighborhoods classified as Very Low or Low SDI, whereas 24% lived in High SDI areas (Supplementary Table S1b).

Median total physical activity was 2431.75 MET-min/week (IQR: 1178.25–4888.25). According to the IPAQ classification, 46.0% of participants were moderately active, 42.4% were highly active, and 11.6% reported low physical activity levels. Smoking and alcohol consumption were common in the cohort, while additional psychological and sleep-related characteristics are summarized in Supplementary Table S1b. Mean total energy intake was 2161±647 kcal/day, with carbohydrates contributing the largest proportion of total energy intake, followed by fat and protein (Supplementary Table S1c).

### 3.1. Identification of Dietary Patterns

The optimal number of clusters was determined using the Elbow method and the Silhouette score, both of which indicated that a three-cluster solution (k = 3) was the most appropriate.

Several internal validation metrics supported this selection: the global silhouette score was 0.110, the Davies–Bouldin index was 2.45, and the Calinski–Harabasz score was 352. The resulting solution explained approximately 17.0% of the total variance (BSS/TSS).

**Figure 3:**
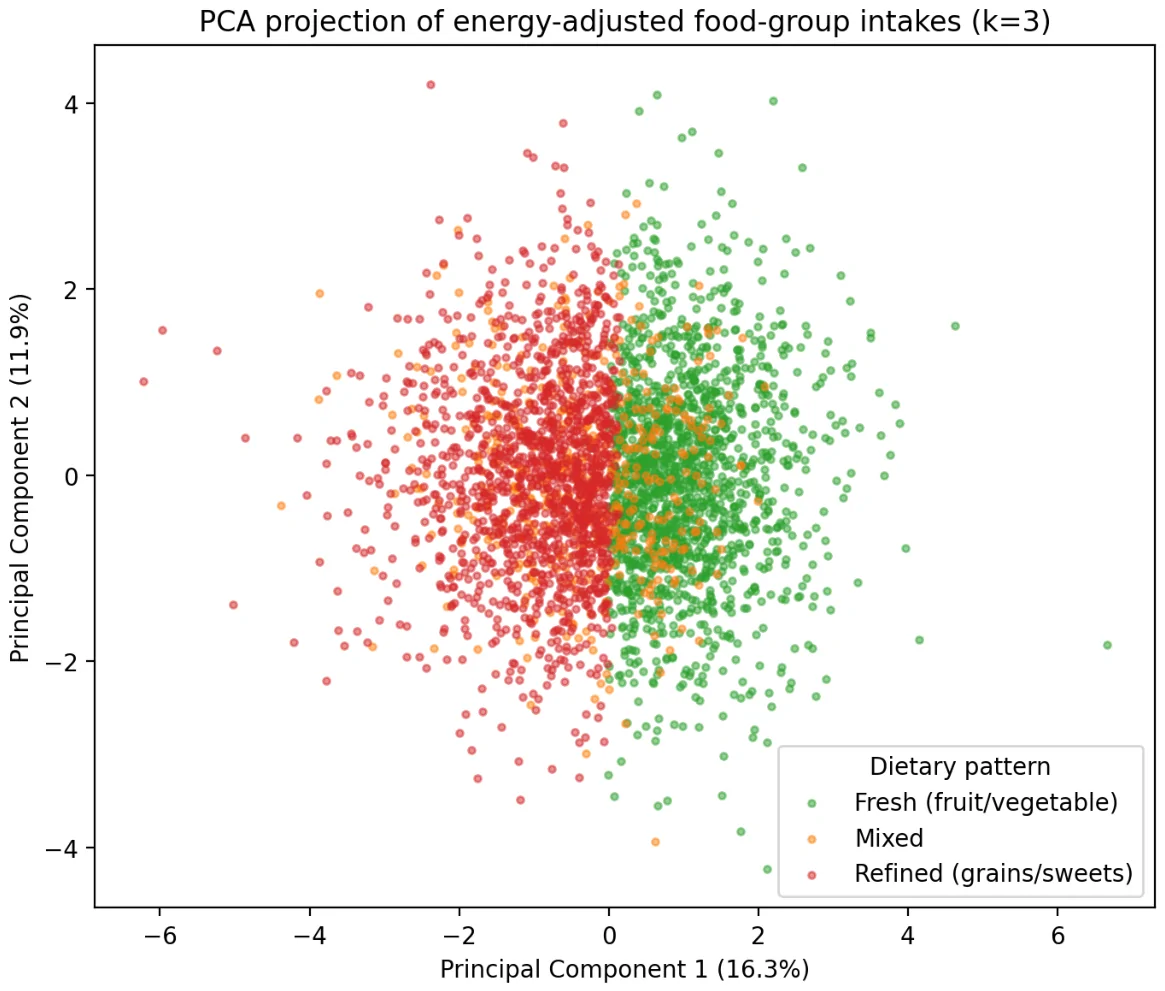
Projection of participants onto the first two principal components (PCA), colored by their k-means cluster (k=3). Each point represents an individual. The PCA was applied for visualization only; clustering was performed on the energy-adjusted, standardized food-group density profiles.

Cluster 1 (Fresh; n = 1530) was characterized by a higher relative contribution of fruits and vegetables. Cluster 2 (Refined; n = 1395) showed a greater contribution of refined grains and sweets. Cluster 3 (Mixed; n = 513) exhibited an intermediate dietary composition, with contributions of fresh and refined food groups falling between those of the Fresh and Refined patterns. For visualization purposes, participants are displayed in the two-dimensional PCA projection (Figure 3); however, cluster assignment was performed using the full set of standardized food-group density variables.

**Figure 4:**
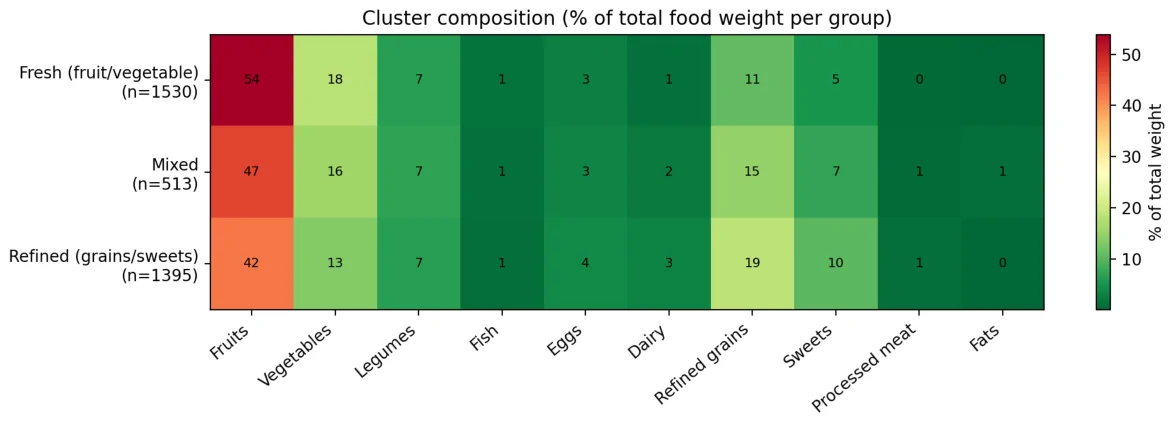
Heatmap of the mean composition of each dietary pattern, expressed as the percentage of total food weight contributed by each food group. Warmer colors indicate a higher relative contribution and cooler colors a lower contribution, as shown by the color bar. Patterns are ordered from the Fresh (fruit/vegetable) to the Refined (grains/sweets) profile. For each participant, the value shown for a food group is its grams divided by the participant’s total food weight (the summed grams across all food groups), expressed as a percentage and then averaged within each cluster. These composition percentages are provided as an intuitive descriptive summary and differ from the variables used for clustering, which were energy-adjusted food-group densities (grams per 1000 kcal), log-transformed and standardized. The heatmap therefore illustrates the relative food-group composition of each pattern rather than the transformed variables on which the clustering was performed. These compositional differences are presented descriptively and were not subjected to formal statistical testing; accordingly, the heatmap should not be interpreted as evidence of statistically significant differences between dietary patterns.

Figure 4 presents a heatmap showing the mean composition (percentage of total food weight per food group) across the three identified dietary patterns. Warmer colors indicate a greater relative contribution of each food group, whereas cooler colors indicate a lower contribution. Fruits represented the primary source of differentiation among patterns: the Fresh pattern showed the highest relative fruit contribution, the Refined pattern the lowest, and the Mixed pattern intermediate values. The Refined pattern was characterized by a greater contribution of refined grains and sweets, whereas the Fresh pattern showed a greater contribution of vegetables. Dairy, fish, processed meat, and fats remained consistently low across all patterns, with only minor differences in their relative contributions among clusters.

**Figure 5:**
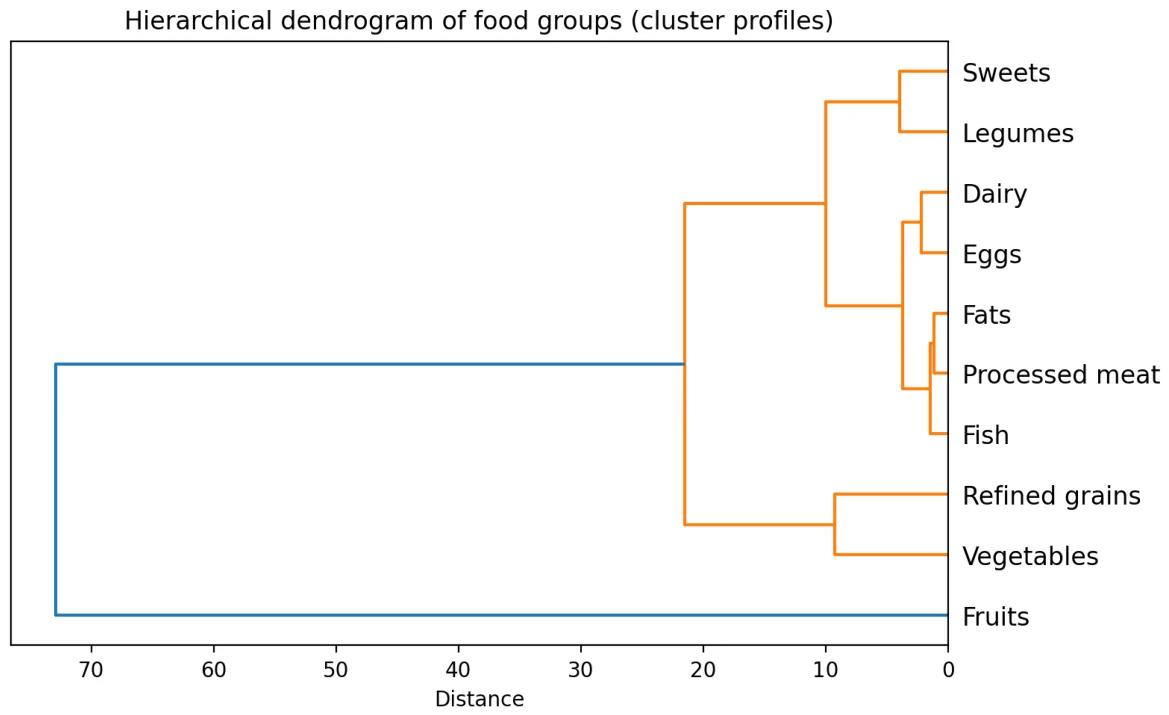
Hierarchical dendrogram of the food groups, based on how similarly each group varies across the three dietary patterns. Note that this dendrogram groups food groups, not participants; the colors are assigned automatically by the linkage distance threshold and do not correspond to the dietary patterns. The horizontal axis represents the distance (dissimilarity) at which groups merge: groups joined at shorter distances vary more similarly across patterns. Fruits form a separate branch because variation in fruit intake contributes most strongly to the dietary gradient identified across the empirical patterns.

To aid interpretation, we constructed a hierarchical dendrogram (Figure 5) that groups food groups according to the similarity of their variation across the three dietary patterns. This aggregated view highlights the food groups that contribute most to pattern differentiation. The most prominent feature is that fruits separate from all other food groups at the largest linkage distance. Among the remaining groups, fresh vegetables and refined grains form the closest pair, whereas dairy, eggs, fish, processed meat, fats, legumes, and sweets cluster together at shorter distances.

Beyond identifying dietary patterns, we next examined whether dietary pattern membership was associated with broader non-dietary characteristics. To this end, the unsupervised clustering framework was complemented with physical activity, sleep quality and psychological stress, cardiometabolic, and sociodemographic variables, which were subsequently evaluated using supervised machine-learning models.

### 3.2. Supervised Classification Performance

During cross-validation, both models achieved a mean balanced accuracy of approximately 0.41. On the independent test set, Random Forest + ADASYN reached an accuracy of 0.528 and a balanced accuracy of 0.413 (macro F1 = 0.383), while HistGradientBoosting + ADASYN achieved an accuracy of 0.508 and a balanced accuracy of 0.403 (macro F1 = 0.383). Across both models, the Fresh and Refined patterns showed higher recall, whereas the Mixed pattern showed the lowest recall. A summary of performance metrics for all supervised classification models is provided in Table 2.

### 3.3. SHAP-Based Interpretation of Dietary Pattern Classification

**Figure 6:**
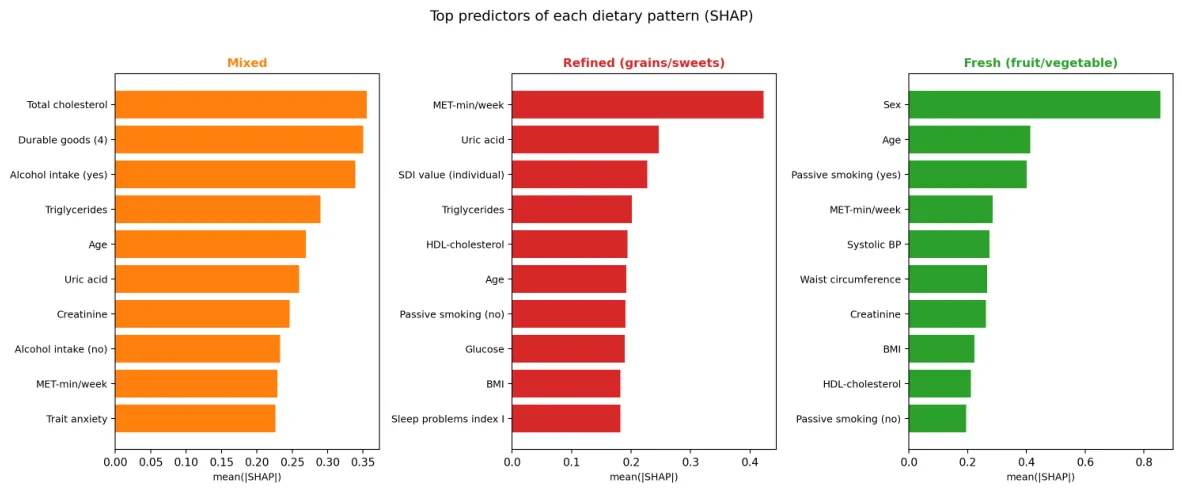
Predictors most associated with each dietary pattern (SHAP analysis of the HistGradientBoosting model). Each panel corresponds to one pattern (Fresh, Refined, Mixed) and shows the ten most influential variables, ranked by mean absolute SHAP value; longer bars indicate a stronger contribution to identifying that pattern. SHAP values reflect learned associations, not causal effects.

SHAP characterizes the variables that most influenced model classification of the empirically derived dietary patterns (Fresh, Refined, and Mixed). Figure 6 summarizes the variables with the largest contributions to the prediction of each dietary pattern.

For the Fresh (fruit/vegetable) pattern, sex was the dominant predictor, followed by age. Female sex markedly increased the model-derived probability of classification into this dietary pattern (mean SHAP =+0.91 for women vs. −0.75 for men), and older age also increased it. Physical activity (MET-min/week), passive smoking status, and several anthropometric and cardiometabolic markers (systolic blood pressure, waist circumference, BMI, and HDL cholesterol) showed smaller contributions to model predictions.

The Refined (grains/sweets) pattern was most strongly associated with physical activity (MET-min/week), followed by cardiometabolic and socioeconomic–territorial features. Higher uric acid and higher triglycerides increased the predicted probability of membership, whereas a higher individual Social Development Index value decreased it. Male sex also increased the predicted probability of this pattern (mean SHAP =+0.37 for men vs. −0.01 for women). The Mixed pattern was most strongly associated with cardiometabolic markers (total cholesterol, triglycerides, uric acid, and creatinine), together with durable-goods ownership and alcohol intake. Taken together, the SHAP analysis showed that different combinations of demographic, lifestyle, anthropometric, cardiometabolic, and socioeconomic–territorial characteristics contributed to the prediction of each dietary pattern.

Table 3 summarizes the distribution of dietary patterns across psycho-behavioral risk categories, derived from the composite index integrating sleep quality, global anxiety, and risk-related behaviors. Pattern distributions were broadly stable across psycho-behavioral risk strata. The Fresh pattern was the most prevalent in the low-risk group (54.9%) and remained the most common in the moderate- and high-risk groups (45.0% and 43.1%, respectively), followed by the Refined pattern (32.5%, 41.1%, and 41.2%) and the Mixed pattern (12.7%, 13.9%, and 15.6%).

### 3.4. Cardiometabolic Biomarkers Across Dietary Clusters

The Fresh pattern showed the lowest median uric acid (5.00 mg/dL), the highest HDL cholesterol (47.6 mg/dL), and the lowest triglycerides (118.4 mg/dL), whereas the Refined pattern showed the highest uric acid (5.52 mg/dL), the lowest HDL cholesterol (44.8 mg/dL), and the highest triglycerides (131.1 mg/dL). The Mixed pattern showed intermediate values for these biomarkers. Sodium and glucose were broadly similar across patterns. Variability was substantial for some biomarkers, particularly triglycerides, resulting in broad distributions within each dietary pattern.

### 3.5. Social Development Conditions Across Dietary Clusters

**Figure 7:**
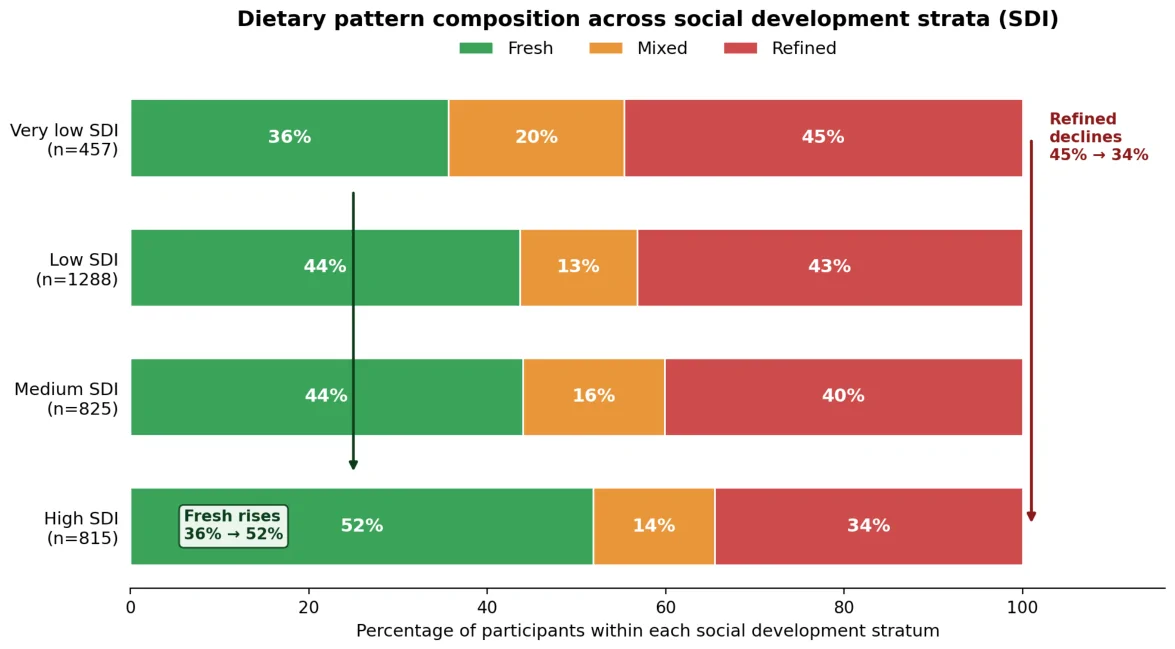
Dietary pattern composition across social development strata. Each bar corresponds to one Social Development Index (SDI) stratum and is divided into the three dietary patterns, summing to 100% within the stratum; percentages and stratum sizes (n) are shown.

Figure 7 shows the composition of dietary patterns within each SDI stratum. The three dietary patterns were observed in every stratum, although their relative frequencies differed across levels of social development. The Fresh pattern increased from 35.7% in the lowest stratum to 51.9% in the highest, whereas the Refined pattern decreased from 44.6% to 34.0% across the same gradient. The Mixed pattern represented between 13.0% and 19.7% of participants across strata. The chi-square test showed a statistically association between SDI stratum and dietary pattern ($\chi^{2}=40.77$, p<0.001), with a Cramér’s V of 0.078.

## 4. Discussion

The present study extends previous analyses from the Tlalpan 2020 cohort by shifting the analytical focus from individual nutrients and cardiometabolic outcomes to empirical dietary patterns as multidimensional behavioral phenotypes, providing a population-level perspective on habitual dietary behaviors and their relationship with the Social Development Index [28]. The analytical sample exhibited anthropometric and cardiometabolic characteristics consistent with well-established sex-related differences in adult populations [60]. Men showed a higher prevalence of overweight and obesity, together with greater waist circumference, blood pressure, triglycerides, LDL cholesterol, and fasting glucose, whereas women presented higher HDL cholesterol concentrations. These characteristics provide important epidemiological context for interpreting the identified dietary patterns and facilitate comparison with other adult cohorts. In addition, over half of the participants resided in neighborhoods classified as Very Low or Low SDI, providing a broad socioeconomic–territorial spectrum within which the relationship between dietary patterns and residential social development could be examined.

Using an integrated machine-learning framework combining unsupervised clustering, supervised characterization, and SHAP-based model interpretation, this study characterizes dietary behavior in relation to the socioeconomic–territorial context through complementary analytical perspectives. The identified dietary patterns primarily reflected variation in the consumption of fresh plant-based foods rather than marked contrasts in the intake of ultra-processed or animal-based foods. As illustrated by the principal component projection (Figure 3), the separation between clusters was modest, consistent with the inherently multidimensional and heterogeneous nature of dietary behavior in complex urban environments [1].

The identified dietary patterns should therefore be interpreted as empirical summaries of predominant dietary compositions along a behavioral continuum rather than as discrete or mutually exclusive dietary phenotypes. This interpretation is consistent with the broader understanding in nutritional epidemiology that data-driven dietary patterns represent approximations of underlying behavioral gradients rather than discrete dietary categories [50,61]. As discussed by Hu [1] and later by Newby and Tucker [62], clustering methods provide useful empirical representations of overall dietary behavior but necessarily simplify a continuous dietary space by assigning each individual to a single pattern. The modest overlap observed between dietary patterns reflects both the inherently continuous nature of dietary behavior and the limited discriminatory capacity of the clustering solution itself, as indicated by the low silhouette score (0.110) and the modest between-cluster variance explained (BSS/TSS ≈17%). Accordingly, the identified patterns are best understood as overlapping dietary gradients rather than well-separated dietary profiles.

Nutritionally, the identified patterns are distinguished by their overall dietary composition rather than by pronounced differences in any single food group. As shown in Figure 4, the Fresh pattern was characterized by a higher relative contribution of fruits and vegetables, whereas the Refined pattern showed a greater contribution of refined grains and sweets together with a lower contribution of fresh plant-based foods. This compositional contrast is consistent with dietary patterns previously identified in nutritional epidemiology, where prudent-type patterns are characterized by higher consumption of fruits and vegetables, whereas Western dietary patterns are associated with greater consumption of refined grains, sweets, and other energy-dense foods [63]. Similar dietary profiles have also been reported in Latin American populations, where Western-type patterns combine higher intakes of refined and processed foods with lower consumption of fruits and vegetables [64,65]. The heatmap in Figure 4 is a descriptive visualization of mean food-group composition within each pattern; the differences shown were not subjected to formal statistical testing and should be interpreted as descriptive rather than inferential.

The hierarchical clustering of food groups (Figure 5) corroborated this nutritional interpretation by showing that fresh plant-based food groups contributed most strongly to the differentiation of dietary patterns, whereas refined grains occupied an intermediate position and the remaining food groups exhibited comparatively smaller contributions to overall dietary variability. The remaining food groups clustered at relatively short distances, indicating a comparatively smaller contribution to dietary differentiation. This pattern of food-group clustering indicates that dietary variability within this cohort was driven primarily by differences in the prominence of fresh plant-based foods, particularly the relative contribution of fresh plant-based foods. This observation is particularly relevant in the Mexican context, where traditional and industrialized foods frequently coexist within the same diet, resulting in hybrid dietary profiles that are not easily captured by conventional dietary classifications [66,67].

A relevant finding of this study was the limited ability of the supervised models to discriminate among dietary patterns. Recall was higher for the Fresh and Refined patterns than for the Mixed pattern, which showed the lowest recall and greater classification overlap with the other two patterns. SHAP analysis provided additional insight into the variables contributing to the prediction of each pattern. For the Fresh pattern, the largest contributions came from sex and age, with female sex and older age increasing the model-predicted probability of membership, consistent with previous evidence that diet quality tends to improve with age and that women report higher fruit and vegetable intake [68]; physical activity and anthropometric and cardiometabolic markers made smaller contributions.

Prediction of the Refined pattern relied more strongly on physical activity, selected cardiometabolic markers, male sex, and lower SDI values, in line with reports linking male sex and higher physical activity levels to less prudent, Western-type dietary patterns [69]; however, this association is not consistent across populations, since other studies have found the opposite direction—greater physical activity associated with healthier, Mediterranean-type patterns rather than Western ones—suggesting that the relationship between physical activity and dietary pattern membership is context-dependent rather than universal.

For the Mixed pattern, the largest contributions came from cardiometabolic variables, alcohol intake, and durable-goods ownership, echoing findings that socioeconomic proxies such as household asset indices are associated with less clearly differentiated or hybrid dietary patterns [70]. Thus, the three patterns were characterized by different combinations of individual and contextual variables rather than by a single consistently discriminating domain.

Differences in cardiometabolic biomarkers across dietary patterns were modest. The Fresh pattern showed lower uric acid and triglyceride concentrations together with higher HDL cholesterol, whereas the Refined pattern showed the opposite tendency. This distribution is compatible with the composition of the identified patterns and with previous evidence linking diets richer in fruits and vegetables to more favorable lipid and metabolic profiles [71,72,73]. Nevertheless, the broad within-pattern distributions observed for several biomarkers indicate that dietary composition alone is unlikely to explain the cardiometabolic heterogeneity of this population.

The association between Social Development Index (SDI) stratum and dietary pattern membership was statistically significant but weak (Figure 7). The proportion of participants classified in the Fresh pattern increased across levels of social development, whereas the Refined pattern became less frequent, in agreement with previous evidence linking socioeconomic conditions to diet quality [74,75]. However, the small Cramér’s V indicates that SDI stratum only weakly differentiated dietary pattern membership within this urban cohort. These findings suggest that residential socioeconomic–territorial conditions represent one component of dietary variation, alongside other individual and contextual influences [76].

Several contextual factors may help explain the modest socioeconomic–territorial gradient observed in this study, although these mechanisms were not directly evaluated. Mexico City’s extensive network of traditional markets (mercados and tianguis), where a large proportion of vendors offer fresh foods [77], may facilitate access to fruits and vegetables across neighborhoods with different levels of social development. In addition, while the SDI captures multiple dimensions of socioeconomic–territorial conditions, it does not directly measure characteristics of the local food environment, such as the availability of food retail outlets, food prices, transportation, or affordability [78,79].

These observations agree with previous research in Mexico City showing that diet quality is more strongly associated with characteristics of the neighborhood food environment than with territorial socioeconomic level alone [79,80]. Similarly, individual-level socioeconomic indicators have been associated with dietary patterns among adults in Mexico City [81], suggesting that both contextual and individual socioeconomic factors may contribute to dietary behavior. Incorporating detailed measures of the food environment into future studies together with individual- and area-level socioeconomic indicators would help clarify the relative contribution of these complementary determinants [82].

This study demonstrates that combining unsupervised clustering, supervised machine learning, and model interpretability techniques provides a richer characterization of dietary behavior than dietary pattern identification alone. Rather than representing discrete dietary phenotypes, the identified patterns describe a continuous spectrum of dietary composition shaped by multiple demographic, behavioral, cardiometabolic, and socioeconomic–territorial influences. This integrated analytical framework may facilitate future investigations of dietary behavior in complex urban populations and support the development of more context-sensitive public health nutrition strategies.

## 5. Limitations

This study has several limitations that should be considered when interpreting its findings. First, the cross-sectional design precludes causal inference. The observed associations between dietary patterns and behavioral, clinical, and socioeconomic–territorial variables represent relationships measured at a single time point and should not be interpreted as temporal or causal. In addition, participants were drawn from a relatively homogeneous urban cohort, in which socioeconomic–territorial variability may be narrower than that observed across broader regional or national populations. Together, these design characteristics may have attenuated the observed association between the SDI and dietary pattern membership.

Second, dietary intake was assessed using a semi-quantitative food frequency questionnaire (SFFQ) with a one-year recall period. Although the instrument has been validated for the Mexican population, self-reported dietary assessment is subject to recall bias and misreporting, which may introduce measurement error. Misreporting has been associated with sex, body weight, educational attainment, and socioeconomic position in Latin American populations [83,84]. Because dietary patterns were derived from the relative composition of food groups rather than absolute intake, the influence of this bias may have been reduced but cannot be ruled out.

Third, participants with a previous diagnosis of hypertension or diabetes were excluded to minimize potential dietary modifications related to disease management, which may limit generalizability to adults living with these common chronic conditions. In addition, dietary data were collected between 2014 and 2019, before the COVID-19 pandemic and subsequent changes in food environments and dietary behaviors. Therefore, the identified patterns should not be interpreted as representing the current distribution of dietary behaviors among adults in Mexico City.

Fourth, the SDI is an area-level measure of socioeconomic conditions based on participants’ place of residence. Consequently, it reflects contextual socioeconomic characteristics rather than individual socioeconomic status. As with any ecological measure, it may not fully capture socioeconomic variability at the individual level. Furthermore, because the SDI does not directly characterize features of the local food environment, such as food retail availability, food prices, transportation, or food affordability, potentially relevant contextual determinants of dietary behavior were not included in the present analyses.

Finally, the analytical framework has inherent methodological limitations. The clustering solution showed modest separation (global silhouette score = 0.110), despite good internal stability (97.5% positive silhouette values; bootstrap adjusted Rand index = 0.93). Accordingly, the identified dietary patterns should be interpreted as empirical representations of a continuous dietary gradient rather than sharply distinct dietary phenotypes. Likewise, the modest predictive performance of the supervised machine-learning models indicates that the variables included in this study explained only part of the variability in dietary pattern membership, suggesting that additional individual, environmental, and contextual determinants contribute to dietary behavior.

## 6. Conclusions

This study identified three empirical dietary patterns in an urban cohort of adults from Mexico City using an integrated machine-learning framework that combined unsupervised pattern identification, supervised characterization, and model interpretability within a socioeconomic–territorial context. Rather than representing sharply distinct dietary phenotypes, the identified patterns reflected a continuous gradient of dietary composition, driven primarily by differences in the relative contribution of fruits, vegetables, refined grains, and sweets, whereas the remaining food groups varied comparatively little across patterns. This gradual compositional shift, rather than sharply distinct profiles, characterizes the dietary variation observed in this cohort.

Although dietary pattern membership was significantly associated with the Social Development Index, the observed socioeconomic–territorial gradient was modest, indicating that residential socioeconomic conditions captured only part of the variability in dietary behavior within this cohort. Similarly, the limited discriminatory performance of the supervised models indicates that dietary behavior reflects the combined influence of multiple individual and contextual factors rather than any single domain of characteristics.

The analytical framework adopted in this study provides a multidimensional characterization of dietary behavior that extends beyond conventional dietary pattern identification. This approach may facilitate future investigations of dietary behavior in complex urban populations and support the integration of individual, environmental, and socioeconomic–territorial dimensions in nutritional epidemiology. Longitudinal studies incorporating detailed measures of the food environment and individual socioeconomic characteristics will be essential to clarify the determinants and long-term health implications of these dietary patterns.

## Figures and Tables

**Table 1:** Operational definition of the composite psycho-behavioral risk index.

Domain	Variable (s)	Original Scale	Categorization Rule
Sleep quality	Sleep duration (SLPQRAW)	Continuous	Lowest tertile (q1 ≤ 6)
	classified as poor sleep		
Anxiety	State and trait anxiety scores	Continuous	High anxiety if either state
	or trait score in the highest tertile		
Behavioral risk	Alcohol intake, energy drink consumption,		
	current smoking, passive smoking exposure	Binary	Presence of at least one risk behavior
Composite index	–	–	Sum of adverse psycho-behavioral
	–	–	domains (range 0–3)

**Table 2:** Supervised classification performance for dietary cluster assignment.

Model	Setting	Accuracy	Balanced Accuracy	Macro F1
Random Forest + ADASYN	Fresh/Refined/Mixed	0.528	0.413	0.383
HistGradientBoosting + ADASYN	Fresh/Refined/Mixed	0.508	0.403	0.383

**Table 3:** Distribution of dietary patterns within each psycho-behavioral risk level. Values are the percentage of participants within each risk level assigned to each dietary pattern (row percentages).

Psycho-Behavioral Risk	Fresh (%)	Mixed (%)	Refined (%)	n
Low	54.9	12.7	32.5	237
Moderate	45.0	13.9	41.1	1028
High	43.1	15.6	41.2	2173

## Data Availability

The raw data supporting the conclusions of this article are not publicly available due to privacy and ethical restrictions, as they belong to the Tlalpan 2020 cohort. Requests to access the datasets should be directed to the corresponding authors.

## Supplementary Materials

The following supporting information can be downloaded at https://www.mdpi.com/article/10.3390/nu18183100/s1. Table S1a. Anthropometric and cardiometabolic characteristics stratified by sex; Table S1b. Sociodemographic and territorial characteristics of the study population; Table S1c. Dietary intake: energy and macronutrient composition.
