# Supplementary material for: Dietary Patterns and the Social Development Index in Mexico City: A Machine-Learning Approach
Source: Nutrients. 2026 Sep 21;18(18):3100. doi: 10.3390/nu18183100 (PMC13610666; doi:10.3390/nu18183100)
Supplement: Supplementary file 1 [file nutrients-18-03100-s001.zip › nutrients-4394402-supplementary.pdf]

nutrients

Article

Dietary Patterns and the Social Development Index in Mexico City: A Machine-Learning Approach

Mireya Martínez-García [1,2,*,†], Guadalupe Gutiérrez-Esparza [3,4,†], María del Carmen González Salazar [5], Luis M. Amezcua-Guerra [1] and Enrique Hernández-Lemus [6,*]

[1] Department of Immunology, National Institute of Cardiology Ignacio Chávez, Mexico City 14080, Mexico; lmamezcuag@gmail.com
[2] Department of Dental Public Health, Graduate Studies and Research Division, School of Dentistry, Universidad Nacional Autónoma de México, Mexico City 04510, Mexico
[3] "Researcher for Mexico" Program Under SECIHTI, Secretariat of Sciences, Humanities, Technology, and Innovation, Mexico City 08400, Mexico; ggutierreze@secihti.mx
[4] Diagnostic and Treatment Services, National Institute of Cardiology Ignacio Chávez, Mexico City 14080, Mexico
[5] Department of Nutrition, Ambulatory Care Services, National Institute of Cardiology Ignacio Chávez, Mexico City 14080, Mexico; carmen.gonzalez@cardiologia.com
[6] Computational Genomics Division, National Institute of Genomic Medicine, Mexico City 14610, Mexico
* Correspondence: mireya.martinez@fo.odonto.unam.mx (M.M.-G.); ehernandez@inmegen.gob.mx (E.H.-L.)
† These authors contributed equally to this work.

1. Supplementary Results

Table S1a. Anthropometric and cardiometabolic characteristics stratified by sex.

Characteristic	Women (n = 2,153)	Men (n = 1,286)
BMI (kg/m^2)	26.2 (23.4–29.7)	27.1 (24.7–30.5)
BMI category, %		
Underweight	1.6	0.6
Normal weight	37.1	27.8
Overweight	37.9	43.4
Obese	23.3	28.2
Waist circumference (cm)	87.0 (79.0–95.0)	94.5 (87.0–102.0)
SBP (mmHg)	104.0 (97.3–112.0)	112.0 (105.3–120.0)
DBP (mmHg)	70.0 (64.0–76.7)	76.0 (70.0–81.3)
Total cholesterol (mg/dL)	179.2 (159.9–202.1)	182.8 (160.9–206.9)
LDL cholesterol (mg/dL)	114.8 (97.5–135.1)	120.8 (101.9–141.2)
HDL cholesterol (mg/dL)	49.8 (42.4–58.5)	41.6 (35.5–47.5)
Triglycerides (mg/dL)	113.1 (82.3–157.6)	146.8 (101.9–211.0)
Fasting glucose (mg/dL)	91.0 (86.0–97.0)	94.0 (89.0–100.0)

Values are median (interquartile range) unless otherwise indicated.
BMI, body mass index; SBP, systolic blood pressure; DBP, diastolic blood pressure; HDL, high-density lipoprotein; LDL, low-density lipoprotein. Men showed higher waist circumference, blood pressure, triglycerides, and lower HDL cholesterol, consistent with expected sex differences in cardiometabolic profile.

 https://doi.org/10.3390/nu18183100

Table S1b. Sociodemographic and territorial characteristics of the study population

Variable	Statistic
General	
Marital status, *n* (%)	
Married	1,675 (48.7)
Single	1,305 (37.9)
Common-law union	335 (9.7)
Divorced / Separated / Widowed	124 (3.6)
Social Development Index (SDI)	
SDI value, mean ± SD	0.8065 ± 0.1058
SDI grade, *n* (%)	
Very Low	454 (13.4)
Low	1,286 (38.0)
Medium	826 (24.4)
High	819 (24.2)
Tobacco use, *n* (%)	
Ever smoked	2,195 (62.8)
Never smoked	1,303 (37.2)
Current smoker	822 (23.5)
Former smoker	698 (20.0)
Passive smoker	1,297 (37.3)
Alcohol and beverage consumption, n (%)	
Alcohol consumption	2,385 (68.2)
Energy drink consumption	272 (8.5)
Physical activity	
Total METs (MET-min/week)	2,431.75 (1,178.25–4,888.25)
IPAQ activity level, *n* (%)	
Low (<600 MET-min/week)	412 (11.6)
Moderate (600–2,999 MET-min/week)	1,640 (46.0)
High (≥3,000 MET-min/week)	1,512 (42.4)
Psychological stress (STAI)	
State anxiety (STAI-S)	30.00 (26.00–35.00)
High anxiety (>45), *n* (%)	210 (6.0)
Trait anxiety (STAI-T)	34.00 (30.00–41.00)
High anxiety (>45), *n* (%)	482 (13.8)
Sleep (MOS Sleep Scale)	
Sleep duration (hours/night), mean ± SD	6.56 ± 1.33
Short sleep (<6 h), *n* (%)	673 (19.2)
Normal sleep (6–9 h), *n* (%)	2,759 (78.9)
Long sleep (>9 h), *n* (%)	66 (1.9)
Sleep adequacy (0–100), mean ± SD	53.96 ± 28.13
Somnolence (0–100), mean ± SD	29.12 ± 16.63
Awakening SOB/headache (0–100)	0.00 (0.00–20.00)
Snoring (0–100), mean ± SD	37.50 ± 31.73
Sleep disturbances (0–100)	21.25 (10.00–36.25)
Sleep Problems Index I (0–100), mean ± SD	30.21 ± 11.55
Global sleep problems, *n* (%)	1,546 (44.2)

Values are median (interquartile range) unless otherwise indicated.
IPAQ, International Physical Activity Questionnaire; MET, metabolic equivalent of task; MOS, Medical Outcomes Study; SOB, shortness of breath; STAI, State–Trait Anxiety Inventory.

Table S1c. Dietary intake: energy and macronutrient composition

Variable	Statistic
Energy	
Total energy intake (kcal/day), mean ± SD	2,161.49 ± 647.17
Macronutrient intake (g/day)	
Total protein	75.84 (61.83–92.52)
Animal protein	40.89 (30.92–52.66)
Total carbohydrates, mean ± SD	276.15 ± 90.47
Sucrose	33.72 (25.46–45.54)
Fructose	22.69 (16.70–30.22)
Fructose net [Frucg(sac-gluc)]	17.64 (13.32–23.82)
Glucose	16.25 (11.65–22.31)
Lactose	7.05 (3.62–11.91)
Maltose	1.26 (0.94–1.68)
Alcohol	1.02 (0.20–1.80)
Dietary fat (g/day)	
Saturated fat	24.52 (19.14–31.07)
Monounsaturated fat	34.58 (27.16–43.96)
Polyunsaturated fat	17.30 (13.59–23.79)
Animal fat	40.91 (30.40–53.96)
Vegetable fat	42.45 (32.41–55.03)
Fat quality ratios	
(Monounsaturated + Polyunsaturated)/Saturated fat ratio	77.94 (61.89–97.45)
Animal/Vegetable fat ratio, mean ± SD	89.43 ± 31.36
Other nutrients	
Sodium (mg/day)	1,679.93 (1,347.94–2,082.89)
Dietary cholesterol (mg/day)	251.37 (192.14–329.05)
Macronutrient distribution (% of total energy)	
Protein	14.47 (12.69–16.27)
Carbohydrates, mean ± SD	51.01 ± 6.72
Fat, mean ± SD	34.17 ± 5.82

Values are median (interquartile range) unless otherwise indicated.
